# Association between Perceived Psychological Stress and Exercise Behaviors: A Cross-Sectional Study Using the Survey of National Physical Fitness

**DOI:** 10.3390/life13102059

**Published:** 2023-10-14

**Authors:** Eun Sun Yoon, Wi-Young So, Seyong Jang

**Affiliations:** 1 Department of Sport for All, College of Educational Sciences, Korea National Open University, Seoul 03087, Republic of Korea; yesjjang3769@knou.ac.kr; 2 Sports Medicine Major, College of Humanities and Arts, Korea National University of Transportation, Chungju-si 27469, Republic of Korea; wowso@ut.ac.kr; 3 Department of Taekwondo, College of Arts and Physical Education, Gachon University, Seongnam 13120, Republic of Korea

**Keywords:** exercise, health behavior, physical activity, psychological stress, survey of national physical fitness

## Abstract

Background: Perceived psychological stress and exercise are bidirectionally related, and the effects of exercise on stress relief are well documented. However, research on the influence of stress on exercise remains scarce. This study examined the association between perceived psychological stress and exercise participation among Korean adults and older adults as well as the relationship between exercise frequency and perceived stress. Methods: Data on 3440 participants (2813 adults aged 19–64 and 627 older adults aged 65 or more) were collected from the Survey of National Physical Fitness conducted by the Korea Institute of Sport Science and the Korean Ministry of Culture, Sports and Tourism in 2015. We compared the participants’ health-related behaviors, including exercise, regular breakfast consumption, and smoking, according to their perceived psychological stress levels. Results: Those who perceived a higher level of psychological stress reported lower levels of exercise participation, regular breakfast consumption, and smoking, with the exception of older male participants. The study also found that a higher frequency of exercise participation corresponded with a lower perceived level of psychological stress (*β* = −0.080, *p* < 0.001) and that engaging in physical activity even once a week yields a substantial reduction in stress levels. Conclusions: In a large sample of Koreans, high levels of perceived psychological stress were significantly associated with less physical activity and infrequent weekly exercise. This study found a dose–response relationship between exercise frequency and reduced stress and suggested that psychological stress should be considered crucial in promoting physical activity.

## 1. Introduction

Psychological stress is common in modern society, and prolonged exposure can affect the cardiovascular, autonomic nervous, and endocrine systems, causing various health problems [1,2,3]. Therefore, it is critical to effectively manage stress and identify coping mechanisms. Exercise is considered an effective method of managing stress. In an initial meta-analysis on fitness and psychological stress response, aerobically fit individuals exhibited a blunted physiological and psychological stress response and faster recovery from stress [4]. Several studies have reported that regular exercise helps reduce stress and can prevent and treat depression, anxiety, and other mental health problems [5,6,7,8]. Thus, many use exercise to relieve stress.

However, although exercise is effective in managing stress, people with high levels of psychological stress tend to adopt a non-physically active lifestyle [9]. Additionally, in stressful situations, individuals tend to engage in unhealthy behaviors, such as smoking, drinking, disordered sleep, irregular eating habits, and infrequent or inadequate physical activity [9,10,11]. While these unhealthy behaviors can offer temporary psychological relaxation, they are considered to be an indirect mechanism through which stress actually deteriorates health [12]. A study reviewing the relationship between stress and exercise also suggested that stress is a predictor of decreased physical activity [13] and is a crucial determinant of exercise behavior. This implies that stress is a significant barrier to achieving the physical activity level required to experience health benefits [14].

Despite the bidirectional relationship between stress and exercise participation, most related studies have focused on the health benefits of exercise. Furthermore, the understanding of the relationship between perceived stress and exercise participation is limited, and exercise participation is often encouraged for individuals irrespective of their perceived stress levels. Many countries have strived to increase their populations’ exercise participation rates by adopting measures like expanding sports facilities, implementing programs, and increasing the number of coaches. However, 80% of adolescents and 27% of adults still do not meet the levels of physical activity recommended by the World Health Organization. Thus, while efforts to change environmental factors to promote physical activity are important, other factors, such as an individual’s psychological state, can also play an important role [15]. Therefore, examining the relationship between perceived stress and exercise behavior is crucial to increase exercise participation rates.

Various studies have also reported sex and age differences in relation to the difference between stress and health behaviors. While women often tend to cope with stress through obesogenic behaviors, such as consuming high-calorie food and resorting to sedentary activities, men often display stress-coping patterns involving non-obesogenic behaviors like increased physical activity or smoking [16,17]. Additionally, stress-coping behaviors are influenced by age. Some research shows that psychological health improves with age [18], and older adults are better at regulating emotional responses to stress than younger individuals [19]. It has further been reported that, even in older adults, stress can be relieved through physical activity, and according to a longitudinal study spanning 4 years, older adults may benefit from improved physical health through stress management [20].

Based on these previous studies, it may be presumed that psychological stress and health behaviors may differ according to gender and age. Therefore, examining the relationship between psychological stress and health behaviors within different groups is expected to be helpful in exploring future targeted strategies to promote healthy behaviors. This study aimed to examine the hypothesis that perceived stress would be associated with health behaviors among healthy Korean adults and older adults. Additionally, we sought to verify the relationship between weekly exercise frequency and stress levels.

## 2. Materials and Methods

### 2.1. Participants

In this cross-sectional study, we analyzed data from the Survey of National Physical Fitness in Korea, collected from 1 October 2015 to 30 November 2015, by the Korea Institute of Sport Science and the Korea Ministry of Culture, Sports and Tourism. This nationwide survey measures physical fitness variables based on sex, age, region, and region size. The appropriate sample size was obtained using the Neyman allocation method, and collective extraction by stratification was used for sample allocation by region. Here, communities were randomly selected by region [21]. Among a total of 4914 participants, we excluded those with missing data on exercise participation and perceived stress. As a result, 3440 participants (1937 men and 1503 women; 2813 adults aged 19–64 and 627 older adults aged 65 or more) were included in our sample. All research procedures were approved by the Korea Institute of Sport Science and the Korean Ministry of Culture, Sports and Tourism. Since the secondary raw data for this study did not include private identifier information, such as telephone numbers, home addresses, name, and social security numbers, ethical approval was not required, and research was conducted based on the principles outlined in the Declaration of Helsinki.

### 2.2. Anthropometric Measurements and Health Behaviors

Height and body weight were measured to the nearest 0.01 cm and 0.01 kg, respectively. Body mass index (BMI) was calculated using the formula BMI = kg/m^2^. A participant was classified as obese when their BMI equaled or exceeded 25 kg/m^2^.

Exercise, average daily sleep time, regular breakfast consumption, and current smoking status were investigated as health behaviors using a self-report questionnaire. The question regarding exercise behaviors was “How many times in a week did you engage in exercise that made you sweat for at least 30 min”. The presence and frequency of exercise participation were also assessed. Participants who responded that they exercised for 30 min at least once a week were defined as exercising participants.

Participants were also asked to provide their typical daily sleep duration with the query, “What is your average daily sleep duration in hours?” In relation to breakfast habits, the participants were asked about their regularity in consuming breakfast using the following response options: “I consistently consume breakfast”, “I occasionally consume breakfast”, “I completely skip breakfast each morning,” “I substitute it with snacks” and “Other”. Participants who indicated that they consistently consumed breakfast were classified as breakfast consumers, while those who provided different responses were categorized as non-breakfast consumers.

Inquiring about smoking habits, participants were presented with the following response choices: “I am presently a smoker”, “I used to smoke in the past, but I do not currently” and “I have never engaged in smoking”. In the context of this study, only individuals who responded “I am presently a smoker” were categorized as current smokers; individuals who had a history of smoking but were presently non-smokers were excluded from this classification. The scientific validity and reliability of these questions (health behaviors and perceived psychological stress) were confirmed by the Survey of National Physical Fitness [21].

### 2.3. Perceived Psychological Stress

Perceived psychological stress was assessed using a self-report questionnaire. The participants were asked to respond to the question presented in the Survey of National Physical Fitness—“On average, how much stress do you feel in your daily life?”—on a 5-point scale, ranging from 1 (“very severe”) to 5 (“very slight”). Those who responded with “very severe” or “severe” were classified into a high-stress group. Those who responded with “moderate” were classified into a moderate-stress group, and those who responded with “slight” or “very slight” were categorized as the low-stress group.

### 2.4. Statistical Analysis

Data were expressed as mean ± standard deviation or number (%). Using a one-way analysis of variance (ANOVA) for continuous and a χ^2^ test for categorical variables, we compared participants’ characteristics based on the three levels of perceived stress (low, moderate, and high). If a significant difference was found among the three groups, a post hoc analysis was performed using Tukey’s range test. Furthermore, we used a simple linear regression analysis to test the effects of exercise frequency on the level of perceived stress. Effect size was calculated as partial eta squared (η^2^p) for the one-way ANOVA, as Cramér’s V for the χ^2^ test, and as R^2^ for the simple linear regression analysis. The effect size of η^2^p was categorized into three levels: large (>0.06), medium (0.01–0.06), and small (<0.01). The effect size of Cramér’s V was categorized into three levels: large (>0.30), medium (0.10–0.30), and small (<0.10). Finally, the effect size of R^2^ was categorized into three levels: large (>0.10), medium (0.01–0.09), and small (<0.01). All data were analyzed using SPSS version 26.0 (IBM Corp.; Armonk, NY, USA), and statistical significance was set at *p* < 0.05.

## 3. Results

### 3.1. Participants’ Characteristics

The average age of the participants was 42.76 ± 16.72 years for men and 42.65 ± 18.80 years for women. The obesity rate was 37.6% for men and 20.8% for women. The level of perceived stress showed a similar distribution among men and women with most participants responding “moderate”. The proportion of exercising participants was higher among men (83.0%) than among women (77.8%), and weekly exercise frequency was similar between men (2.66 ± 2.01 times a week) and women (2.56 ± 2.00 times a week). The proportion of those who consumed breakfast regularly was higher among women (54.1%) than among men (50.5%), and the proportion of current smokers was higher among men (26.9%; Table 1).

### 3.2. Comparison of Health Indices Based on the Level of Perceived Stress

Among men, 18.1%, 56.4%, and 25.5% perceived low, moderate, and high levels of stress, respectively. These percentages were 18.2%, 58.9%, and 22.9%, respectively, among women, indicating a similar distribution of perceived stress levels between the two groups. While there were no differences in age among men according to their perceived stress levels, younger women perceived stress more negatively (F = 18.218, *p* < 0.001, η^2^p = 0.031). Although there were significant differences in BMI among both men and women based on their perceived stress levels, the pattern differed between the two sexes. Men who perceived a high level of stress had higher BMI, whereas women who perceived a high level of stress had a lower BMI. Men who perceived high-stress levels showed higher obesity rates (χ^2^ = 8.816, *p* = 0.001, Cramér’s V = 0.072), and no significant differences were seen in obesity rates among women based on the level of their perceived stress.

As the perceived stress level increased, regular exercise at least once a week (men, χ^2^ = 23.058, *p* < 0.001, Cramér’s V = 0.117; women, χ^2^ = 17.206, *p* < 0.001, Cramér’s V = 0.123) and exercise frequency per week (men, F = 18.360, *p* < 0.001, η^2^p = 0.022; women, F = 13.930, *p* < 0.001, η^2^p = 0.024) significantly decreased in both men and women. Additionally, both men and women reported lower rates of regular breakfast consumption (men, χ^2^ = 28.068, *p* < 0.001, Cramér’s V = 0.129; women, χ^2^ = 18.132, *p* < 0.001, Cramér’s V = 0.113) and higher rates of smoking with increasing stress levels (men, χ^2^ = 15.458, *p* = 0.003, Cramér’s V = 0.068; women, χ^2^ = 19.005, *p* < 0.001, Cramér’s V = 0.091). The average duration of daily sleep decreased as the level of perceived stress increased, but statistically significant differences were observed only among men (F = 2987.458, *p* < 0.001, η^2^p = 0.129; Table 2).

Regarding older adults, no significant differences existed in the age, BMI, and obesity rates of either sex based on their perceived stress levels. Higher levels of perceived stress were significantly associated with less exercise participation (χ^2^ = 6.518, *p* = 0.038, Cramér’s V = 0.134) and exercise frequency (F = 107.691, *p* < 0.001, η^2^p = 0.047) among older women. However, no significant differences were found in their sleep duration, regular breakfast consumption, and smoking practice based on their perceived stress level. Among older men, those who perceived their stress level as “moderate” had the lowest exercise frequency, the shortest sleep duration, and the lowest rate of regular breakfast consumption. However, no significant differences existed in their exercise participation based on their perceived stress level (Table 3).

### 3.3. Relationship between Exercise Frequency and Perceived Stress Levels

**Figure 1:**
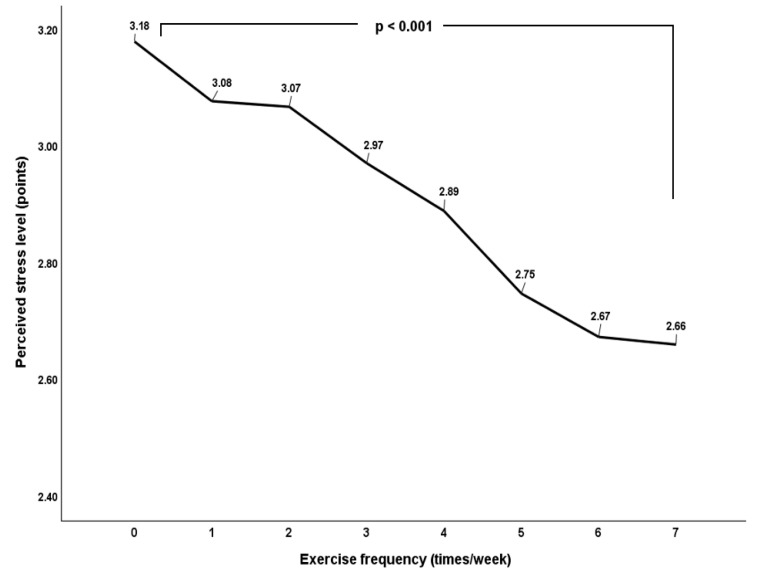
Perceived stress level based on exercise frequency.

Exercise frequency was found to affect an individual’s perceived stress. Simple linear regression analysis revealed a significant relationship between exercise frequency and perceived stress with higher exercise frequency being associated with lower levels of perceived stress. Furthermore, the one-way ANOVA revealed that even those who exercised only once a week had significantly lower levels of perceived stress. There were no significant differences in perceived stress based on exercise frequency for those who exercised five or more times a week (Table 4; Figure 1).

## 4. Discussion

This study aimed to examine the hypothesis that perceived stress and health behaviors are related among Korean adults and older adults. The research findings indicated a significant negative association between perceived stress levels and regular exercise participation and weekly exercise frequency with higher stress corresponding to lower levels of regular exercise in all groups except for older men. Furthermore, among adult men and women, higher levels of perceived stress were associated with engaging in unhealthy behaviors, including shorter sleep durations, irregular breakfast consumption, and higher smoking rates. However, the relationship between perceived stress levels and health behaviors was not clearly evident in older men. Furthermore, an analysis of the differences between weekly exercise participation frequency and perceived stress scores revealed that higher weekly participation frequency was significantly associated with lower levels of perceived stress. Interestingly, even participating in exercise only once a week was associated with a significant reduction in perceived stress scores compared to not engaging in any exercise at all.

The findings of this study support similar previous studies of associations between psychological stress and health behavior, including exercise participation [11,22,23]. For example, a study conducted in Denmark involving 8835 participants found that perceived stress was significantly associated with low intake of fruit or vegetables, daily smoking, and physical inactivity [22]. Previous studies about the association between perceived stress and physical inactivity have reported that negative emotions, such as stress and depression, significantly increase sedentary behavior and reduce physical activity, and stress is associated with reduced physical activity and sedentary behaviors [13,24,25,26]. Thus, although exercise effectively reduces negative emotions, such as stress, people are more likely to adopt a sedentary lifestyle in stressful situations, resulting in less exercise participation.

Interestingly, stress not only reduces exercise participation, but also affects exercise satisfaction and early dropout rates. Studies similar to this one have focused on identifying effective interventions to prevent decreases in physical activity among college students. One study found that perceived stress is most strongly related to physical activity participation and suggested that providing guidelines on stress management and physical activity together could be an effective strategy for promoting physical activity [25].

Similar results have been reported for various groups with different health conditions. A prior study on the effectiveness of exercise and medication therapy programs for people diagnosed with depression found that preexisting negative emotions, like anxiety and low life satisfaction, were the strongest predictors of dropout and low treatment outcomes [27]. Similar results were found for cardiac rehabilitation programs [28]. One study involving an obesity treatment program found that positive emotional states in the morning increased the likelihood of participating in exercise that day. It also found that higher exercise intensity and duration were associated with higher positive emotions, and enhancing positive emotions was related to more exercise initiation and higher exercise intensity. The study suggested that assessing participants’ emotions before and after the exercise program could provide vital information for promoting physical activity [29].

The mechanism underlying the negative effect of stress on exercise participation has not yet been determined. However, according to Nigg et al.’s [30] theory of physical activity maintenance, stress can directly or indirectly promote or hinder physical activity. Stress reduces the motivation to engage in an active lifestyle and can increase feelings of depression, anxiety, physical weakness, and fatigue, leading to decreased physical activity. Moreover, people tend to lack self-regulatory resources when under stress, which can induce them to avoid physical activity and choose unhealthy, emotion-focused coping strategies, such as smoking, drinking, and overeating [31,32].

In this study, adult men who perceived higher stress levels had lower rates of exercise participation and regular breakfast consumption but higher rates of smoking and obesity. Similarly, the Korean Statistical Information Service’s social survey reported that, as stress levels increase, people tend to engage less in healthy behaviors, such as health checkups, breakfast consumption, adequate sleep, and exercise. Among these, exercise participation is the most affected by increased stress levels [33].

Recently, questions have been raised regarding the amount of exercise required to benefit mental health and its relationship with stress. In this study, we identified a dose–response relationship between exercise frequency and reduced stress levels. We found that even exercising once a week significantly decreased stress levels compared to not exercising. These results are consistent with previous studies showing that even low or moderate daily activity can effectively reduce stress [34] and more frequent exercise is associated with fewer depressive symptoms [35].

However, more exercise does not always lead to better results. A study analyzing the relationship between exercise and mental health over a month among 1.2 million American adults found that exercise frequency and duration had a nonlinear relationship with mental health. The relationship was U-shaped; people who exercised 3–5 times a week had the best mental health status, whereas those who exercised more than 23 times a month for 90 min or more per session had a negative mental health status [36]. However, as shown in this study, it is noteworthy that exercising even up to three times a week significantly improved the mental health of exercisers over non-exercisers, suggesting that even a small amount of exercise is better than no exercise for maintaining good mental health. Additionally, an observational study with over 30,000 individuals found that even just one hour of exercise a week leads to continuous improvement in depressive symptoms [7], indicating that physical activity even below the recommended amount can positively contribute to reducing stress.

The current study had several limitations. First, as a cross-sectional study, it could not prove the causality between changes in stress and exercise behavior. Second, this study only assessed the level of perceived stress and thus could not identify the causes and degree of stress. This made it difficult to clearly identify changes in exercise behavior based on different stressors. However, since this study investigated people’s tendency to perceive stress in daily life, it could provide data on the cognitive interpretation of stress and its relationship with exercise.

Third, this study did not investigate the type, duration, or intensity of exercise. However, it aimed to clarify how stress affects exercise participation, not the role of exercise in stress management. According to Chekroud et al. [36], whose study involved 1.2 million people, exercise positively contributes to improving mental health regardless of its type or intensity. Therefore, further in-depth research is needed to investigate the relationship between stress and specific exercise variables measured using objective physical activity monitoring devices, such as wearable devices.

Fourth, this study targeted healthy adults and did not investigate the impact of illness. Fifth, differences were expected between genders and age groups in health behaviors caused by stress. However, in this study, due to the difference in the number of adults under 65 years of age and seniors over 65 years, these groups were not compared; instead, groups were compared separately. Sixth, this study did not use a validated questionnaire for stress determination, such as Perceived Stress Questionnaire; therefore, the results might be not reliable since they are based on individual opinions and perceptions. For this reason, in the future, further well designed and validated studies are necessary. Despite these limitations, this study is significant because it analyzed the relationship between stress and exercise using a representative nationwide sample.

A more in-depth exploration of the relationship between stress and exercise is considered a fundamental endeavor, and the following additional research is deemed necessary in this regard. Initially, a more detailed investigation is needed into the relationship between the type, intensity, and duration of physical activity and stress levels to study the specific exercise factors influencing stress reduction. Furthermore, longitudinal observational and experimental studies concerning the long-term associations among stress, exercise participation, and health outcomes are required. These research findings are expected to be utilized in the future in the development of programs aimed at effectively enhancing exercise participation rates.

## 5. Conclusions

This study shows that higher levels of perceived psychological stress are significantly associated with unhealthy behaviors. Additionally, a dose–response relationship was observed between exercise frequency and stress levels. These results suggest that psychological stress should be considered a crucial factor when planning physical activity promotion programs. Furthermore, additional experimental and longitudinal observational studies are needed in the future to achieve a deeper understanding of the relationship between stress and exercise.

## Figures and Tables

**Table 1:** Participants’ characteristics.

Variables	Men	Women
n	1937	1503
Age (years)	42.76 ± 16.72	42.65 ± 18.80
Height (cm)	172.12 ± 6.53	158.55 ± 6.23
Weight (kg)	72.24 ± 9.76	56.91 ± 7.59
Body mass index (kg/m^2^)	24.36 ± 2.80	22.67 ± 3.01
Obesity (n, %)	727 (37.6%)	313 (20.8%)
Perceived stress level (n, %)		
Low	397 (20.5%)	314 (20.9%)
Moderate	1083 (55.9%)	879 (58.5%)
High	457 (23.6%)	310 (20.6%)
Exercise participation at least once a week (n, %)	1608 (83.0%)	1170 (77.8)
Exercise frequency (times/week)	2.66 ± 2.01	2.56 ± 2.00
Sleep (hours/day)	6.58 ± 1.08	6.60 ± 1.14
Regular breakfast consumption (n, %)	977 (50.5%)	813 (54.1%)
Currently a smoker (n, %)	522 (26.9%)	32 (2.1%)

**Table 2:** Characteristics of adults (aged between 19 and 64) based on the perceived stress level.

Variable	Men (n = 1674)					Women (n = 1139)				
	Low	Moderate	High	*p*	Effect Size	Low	Moderate	High	*p*	Effect Size
N (%)	303 (18.1%)	944 (56.4%)	427 (25.5%)			207 (18.2%)	671 (58.9%)	263 (22.9%)		
Age (years)	38.90 ± 14.20	38.16 ± 12.76	37.51 ± 11.42	0.329	0.001	43.43 ± 13.27	39.29 ± 12.85 ^a^	36.27 ± 12.71 ^a,b^	<0.001 ***	0.031
Height (cm)	172.25 ± 6.56	173.34 ± 5.98 ^a^	173.23 ± 6.02	0.024 *	0.004	159.34 ± 5.32	160.10 ± 5.72	160.50 ± 5.83	0.074	0.005
Weight (kg)	71.37 ± 8.98	73.39 ± 9.81 ^a^	73.90 ± 9.79 ^a^	0.001 **	0.008	57.67 ± 7.56	56.98 ± 7.49	56.47 ± 8.36	0.234	0.003
Body mass index (kg/m^2^)	24.05 ± 2.68	24.41 ± 2.88	24.61 ± 2.90 ^a^	0.036 *	0.004	22.74 ± 2.92	22.25 ± 2.87	21.94 ± 3.17 ^a^	0.012 *	0.008
Obesity (n, %)	92 (30.5%)	376 (39.9%)	170 (39.8%)	0.010 *	0.072	38 (18.4%)	102 (15.2%)	42 (16.1%)	0.555	0.036
Exercise participation at least once a week (n, %)	261 (86.1%)	792 (83.9%)	317 (74.2%)	<0.001 ***	0.117	175 (84.5%)	497 (74.1%)	177 (67.8%)	<0.001 ***	0.123
Exercise frequency (times/week)	2.97 ± 1.96	2.46 ± 1.88 ^a^	2.11 ± 1.88 ^a,b^	<0.001 ***	0.022	2.81 ± 1.94	2.18 ± 1.87 ^a^	1.92 ± 1.77 ^a^	<0.001 ***	0.024
Sleep (hours/day)	6.86 ± 1.03	6.62 ± 1.04 ^a^	6.36 ± 1.03 ^a,b^	<0.001 ***	0.024	6.81 ± 1.15	6.73 ± 1.05	6.58 ± 1.11	0.067	0.005
Regular breakfast consumption (n, %)	177 (58.6%)	429 (45.5%)	165 (38.6%)	<0.001 ***	0.129	113 (54.6%)	325 (48.5%)	100 (38.3%)	<0.001 ***	0.113
Current smoker (n, %)	66 (21.8%)	269 (28.5%)	148 (34.7%)	0.003 **	0.068	2 (1.0%)	9 (1.3%)	13 (5.0%)	0.001 **	0.091

* *p <* 0.05, ** *p <* 0.01, *** *p <* 0.001; tested using one-way analysis of variance with Tukey’s post hoc and χ^2^ tests. ^a^ *p* < 0.05 vs. low-stress group. ^b^ *p* < 0.05 vs. moderate-stress group. Effect size was calculated as partial eta squared for the one-way analysis of variance and as Cramér’s V for the χ^2^ test.

**Table 3:** Characteristics of older adults (aged over 65) based on the perceived stress level.

Variable	Men (n = 263)					Women (n = 365)				
	Low	Moderate	High	*p*	Effect Size	Low	Moderate	High	*p*	Effect Size
N (%)	94 (35.7%)	139 (52.9%)	30 (11.4%)			107 (29.4%)	208 (57.1%)	49 (13.5%)		
Age (years)	72.37 ± 5.33	72.40 ± 5.20	70.70 ± 5.17	0.255	0.010	73.75 ± 5.42	73.79 ± 6.06	72.43 ± 5.98	0.326	0.006
Height (cm)	165.07 ± 4.85	166.25 ± 5.92	166.06 ± 5.50	0.268	0.010	153.71 ± 5.22	153.99 ± 5.75	153.59 ± 5.01	0.857	0.001
Weight (kg)	65.46 ± 7.22	66.89 ± 8.25	67.14 ± 8.91	0.355	0.008	57.20 ± 6.48	56.32 ± 7.28	56.97 ± 8.34	0.565	0.003
Body mass index (kg/m^2^)	24.02 ± 2.42	24.17 ± 2.42	24.29 ± 2.57	0.835	0.001	24.23 ± 2.69	23.75 ± 2.79	24.15 ± 3.32	0.311	0.006
Obesity (n, %)	32 (34.0%)	48 (34.5%)	9 (30.0%)	0.892	0.030	47 (43.9%)	64 (30.8%)	20 (40.8%)	0.053	0.124
Exercise participation at least once a week (n, %)	88 (93.6%)	122 (87.8%)	28 (93.3%)	0.280	0.098	101 (94.4%)	180 (86.5%)	40 (81.6%)	0.038 *	0.134
Exercise frequency (times/week)	4.50 ± 2.14	3.52 ± 2.07 ^a^	3.97 ± 2.22	0.003 **	0.045	4.24 ± 1.90	3.30 ± 2.04 ^a^	3.16 ± 2.11 ^a^	<0.001 ***	0.047
Sleep (hours/day)	6.73 ± 1.24	6.25 ± 1.20 ^a^	6.60 ± 1.25	0.011 *	0.034	6.22 ± 1.17	6.26 ± 1.26	6.29 ± 1.22	0.947	0.001
Regular breakfast consumption (n, %)	84 (89.4%)	106 (40.3%)	16 (53.3%)	<0.001 ***	0.263	82 (76.6%)	162 (77.9%)	31 (63.3%)	0.096	0.114
Current smoker (n, %)	7 (7.4%)	26 (18.7%)	6 (20.0%)	0.079	0.126	4 (3.7%)	3 (1.4%)	1 (2.0%)	0.523	0.067

* *p <* 0.05, ** *p <* 0.01, *** *p <* 0.001; tested using one-way analysis of variance with Tukey’s post hoc and χ^2^ tests. ^a^ *p* < 0.05 vs. low-stress group. Effect size was calculated as partial eta squared for the one-way analysis of variance and as Cramér’s V for the χ^2^ test.

**Table 4:** Effect of exercise frequency on perceived stress.

Variable	Unstandardized Coefficients		Standardized Coefficients	*t*(*p*)	*F*(*p*)	*R* ^2^
	Beta	Standard Error	*β*			
(Constant)	3.188	0.023		137.166 ***	127.735 ***	0.036
Exercise frequency	−0.080	0.007	−0.189	−11.302 ***		

*** *p <* 0.001; tested using simple linear regression analysis.

## Data Availability

The data presented in this study are available upon request from the authors. Some variables were restricted to preserve the anonymity of study participants.
